# Phosphorylation of intrinsically disordered regions steers the protein-RNA binding profiles

**DOI:** 10.1016/j.tig.2024.04.004

**Published:** 2024-05-04

**Authors:** Miha Modic, Maksimiljan Adamek, Jernej Ule

**Affiliations:** 1 National Institute of Chemistry, Ljubljana, Slovenia; 2 The Francis Crick Institute, London, United Kingdom; 3 UK Dementia Research Institute at King’s College London, London, United Kingdom; 4 PhD Program ‘Biosciences’, Biotechnical Faculty, University of Ljubljana, Ljubljana, Slovenia

## Abstract

Due to their capacity to mediate repetitive protein interactions, intrinsically disordered regions (IDRs) are crucial for the formation of various types of protein-RNA complexes. The functions of IDRs are strongly modulated by post-translational modifications. Phosphorylation is the most common and well-studied modification of IDRs, which can alter homomeric or heteromeric interactions of proteins, and impact their ability to phase-separate. Moreover, phosphorylation can influence the RNA-binding properties of proteins, and recent studies demonstrated its selective impact on the global profiles of protein-RNA binding and regulation. These findings highlight the need for further integrative approaches to understand how signalling remodels protein-RNA networks in cells.

## IDRs are responsive to cellular signalling

RNA-binding proteins (RBPs) are central players in post-transcriptional gene regulation. Our knowledge of their regulatory mechanisms is growing rapidly, including the increasing understanding of how their activity is controlled by cellular signalling pathways. Signalling pathways are key to the dynamic regulation of cellular functions, usually through reversible post-translational protein modifications (PTMs) in their ability to rapidly reprogram protein functions. While many studies have focused on PTMs acting as on/off switches for pathway implementation [1], it is increasingly recognised that PTMs can also tune regulatory layers by modulating intra- and intermolecular interactions and altering the conformation of intrinsically disordered regions (IDRs) (Figure 1, left column). Recent proteomic studies have systematically catalogued the PTMs in RBPs that are enriched in IDRs [2–4]. While phosphorylation sites are the most prevalent, acetylation, ubiquitination and methylation sites are also found in almost half of the RBPs [2,5]. IDR phosphorylation has been the focus of far more studies than other PTMs in RBPs. Therefore, this review will focus on the studies of phosphorylation sites in the IDRs of RBPs and the insights we have gained into their role in the reorganisation of protein-RNA interactions across the transcriptome.

IDRs exist in a collection of rapidly interconverting, structurally diverse conformations called an ensemble. Despite the lack of a stable structure, intramolecular contacts create sequence-specific structural biases that determine ensemble properties and the functionalities of the IDRs [6]. PTMs can modify such intramolecular contacts to change the bias of IDRs towards certain conformations or ensemble properties, depending on the type of the PTM and its positioning within the IDR [7]. Short IDRs located between folded domains are commonly phosphorylated to induce protein conformational changes, as is the case for kinase activation loops [8]. It remains to be seen if phosphorylation affects IDR linkers that are located between RNA binding domains such as RNA-recognition motifs (RRMs), which upon RNA binding become partly structured and thereby assist in the rearrangement of RRMs [9]. For longer IDRs, phosphorylation tends to elongate the conformations of neutral or overall negatively charged IDRs, whereas for IDRs containing clusters of positively charged residues, it can lead to chain compaction due to electrostatic interactions between phosphorylated residues and the positively charged clusters [10]. Largely due to these effects of PTMs, IDR conformations and functions are highly responsive to the cellular signalling and chemical environment [11].

## The impact of IDR phosphorylation on RNP assembly

The impact on local protein structures and disorder-to-order transition has been most well documented for phosphorylation [12,13] (Figure 1, left column). IDR phosphorylation can stabilise and destabilise transient helices in a position-dependent manner [14], for example by inducing a more compact conformational ensemble in the IDR of PAGE4 [15]. Similarly, phosphorylation of the transcription factor Ets1 promotes folding of the intrinsically disordered serine-rich region (SRR) into a 3_10_-helix, which in turn forms new interactions with the protein core. This new interaction greatly reduces the DNA binding activity of Ets1 [16]. An even more dramatic structural rearrangement is induced by phosphorylation of two threonines in the IDR of 4E-BP2, which induce its folding into a four-stranded β-domain. This folded form sequesters a motif of 4E-BP2 that otherwise binds to eIF4E, and thus phosphorylation reduces the 4E-BP2-eIF4E affinity by a factor of approximately 4,000 [17–19].

Most studies linking structural and functional changes in IDRs have been conducted *in vitro*, but recent evidence suggests that these structural biases also exist *in vivo*, where the intramolecular interactions can dynamically respond to cellular environment changes, reshaping their structural properties [6,20]. In a recent study, proteomic and bioinformatic information was mapped onto AlphaFold2 3D models to identify structural fingerprints of modifications. This revealed that different regulatory PTMs are usually close to each other in 3D and not only in sequence space, hinting at their mutual influence [8].

The importance of IDRs in all aspects of RBP functions is well recognised and has been thoroughly reviewed [21]. Genome-wide RNA interactome capture assays have identified hundreds of proteins that crosslink to mRNA but lack structured RNA-binding domains. Peptides crosslinked to RNA are frequently assigned to IDRs, suggesting that IDRs contribute to the assembly of a broad range of ribonucleoprotein complexes (RNPs) [22]. IDRs are capable of mediating repetitive (and usually weak) homomeric and heteromeric protein interactions and can also contact RNA [23]. However, a recent study suggested that proteins lacking structured RNA-binding domains generally form only weak and nonspecific interactions with RNA [24]. Phosphorylation of IDRs can affect the switching between monomer and dimer forms [25], the homomeric assembly of an RBP into a regular structural unit [26], or the IDR-mediated condensation of heterogeneous RNPs [27,28]. Thus, IDR phosphorylation can enhance or suppress attractive interactions that modulate phase separation propensity and thus the formation of RNP condensates [29,30].

## The impact of IDR phosphorylation on RNP functions

With increasing understanding of the effects of PTMs on the assembly of many types of RNPs *in vitro* and *in vivo*, it is also becoming possible to characterise the cellular functions of PTMs in RBPs [3] (Figure 1, middle column). In the case of FMRP, phase separation promoted by phosphoregulation leads to its sequestration into RNP condensates that repress translation of bound RNA [31,32]. Moreover, cellular functions of RBPs as mediators of signalling have been studied especially well in the context of alternative splicing regulation, where IDRs such as the arginine-serine (RS) domain in SR proteins were found to be the hotspot for PTMs [33,34]. RS-domain phosphorylation was found to induce a conformational switch from a disordered state to a partially rigidified arch-like structure that facilitates interactions with specific proteins, while also non-specifically interacting with RNA [35]. These structural changes likely contribute to a functional switch: phosphorylated RS domains promote recruitment of SR proteins to transcription sites and assembly of the spliceosome, whereas dephosphorylated SR proteins generally promote the formation of a fully active spliceosome capable of splicing catalysis as well as mRNP packaging and nuclear export [36].

Another well-studied phosphorylated RBP is G3BP1, which contains three IDRs, such that phosphorylation of IDR1 promotes autoinhibitory interactions between IDR1 and IDR3 that compete with RNA binding [37]. Thus, IDR1 phosphorylation impairs the ability of G3BP1 to perform liquid-liquid phase separation with RNA and inhibits its role in stress granule assembly. Similarly, IDR phosphorylation of SAM68 or RBPMS decreases their oligomerization propensity, which reduces their ability to bind RNA [38,39]. Phosphorylation of IDRs has thus been often linked to reduced capacity of RBPs to interact with RNA or with other proteins [40]. The reduced binding capacity can be position-dependent as phosphorylation sites that are close to RNA-binding regions tend to be more inhibitory [21], possibly due to charge-charge repulsions between the phosphoryl group and polyelectrolytes like RNA that are both negatively charged.

While common principles are emerging from studies of various phosphorylated RBPs, proteomic studies have also systematically investigated the effects of IDR phosphorylation on protein-RNA interactions in living cells. In a recent proteomic study, the phosphorylation sites with potential impact on RNA binding were investigated, identifying nearly 200 phosphorylation sites that alter mRNA binding capacity by at least twofold. Surprisingly, 86 sites were associated with increased RNA binding and 102 sites with decreased RNA binding, suggesting that phosphorylation can cause diverse types of changes. The effects on RNA binding could result from altered RBP localisation; for example, several phosphorylation sites were identified in the C-terminal disordered region of RBM20 that affect its nucleocytoplasmic localisation [3].

## IDR phosphorylation can alter the RNA binding profiles

In addition to the general effects of PTMs on the ability of an RBP to bind RNA, the next challenge is to characterise how specific PTMs can modulate the interaction of a protein with a particular set of RNA partners. In a recent study, iCLIP (individual-nucleotide resolution CrossLinking and ImmunoPrecipitation) was used to investigate the transcriptome-wide RNA binding profile of LIN28A at multiple stages during the transition to pluripotent cells. In particular, it was shown that MEK-dependent phosphorylation of the LIN28A IDR leads to a comprehensive restructuring of its RNA interactions. Phosphorylated LIN28A (pLIN28A) binds less to its characteristic WGG-rich motifs and binds more to multivalent AUU-rich motifs in the terminal regions of the 3’UTRs of a specific group of mRNAs associated with naïve pluripotency [41]. This explains how pLIN28A triggers selective mRNA decay to ensure elimination of the naive expression programme required for the transition to primed pluripotency. Remarkably, iCLIP also identified strong binding of polyadenylate-binding proteins (PABPs) to the terminal regions of naïve pluripotency-associated mRNAs, which is enhanced by the convergent binding of pLIN28A to the same regions (Figure 1, right column). This suggests that PABP not only binds polyadenylation tails (poly(A)), but also contacts multivalent termini of specific 3’UTRs that serve as hubs for signal-induced binding of additional RBPs, such as LIN28A. PABP generally suppresses decay by binding to poly(A) tails, protecting them from deadenylation [42]. It has also been shown capable of binding 3’UTR sequences to promote mRNA decay by activating CCR4-NOT complex that induces deadenylation [43,44]. This raises an intriguing possibility that PABP may switch between opposing regulatory modes in response to signal-induced convergence with pLIN28A at the AUU multivalent termini of specific mRNAs. Thus, it is clear that protein PTMs can alter RNA binding and regulation in a highly selective manner.

Studies on IDRs in RBPs are beginning to converge with studies on the role of RBPs in cellular signalling, but many questions remain unanswered. For example, IDR-mediated condensation of TDP-43 promotes efficient binding to long multivalent RNA regions with dispersed binding motifs, but whether post-translational modifications of TDP-43 lead to similar effects remains to be examined [45]. Both the N- and C-terminal regions of TDP-43 are subject to phosphorylation and many other PTMs. Some of these PTMs have been shown to modulate phase separation and RNA binding in ways that may be relevant to the function of TDP-43 or to the pathology of neurodegenerative diseases [30,46,47]. Similarly, selective impact of PTMs on RNA binding likely takes place in many hnRNPs where IDRs contribute to multivalent assembly on RNAs [38,39,48,49]. Conversely, many RBPs have been found to respond to cellular signals and play a role in biological transitions, but it remains to be seen how protein-RNA interactions are altered to rewire regulatory networks. For example, *in vivo* studies revealed that many RBPs change their absolute amount of bound RNA in response to cell fate transition or IDR phosphorylation [3,41], but for most of these, selectivity of changes in binding profiles to specific RNAs still needs to be examined.

## Future directions

Ultimately, it must be clarified how signal transduction affects the assembly or condensation of several proteins on long RNA molecules. Another question that needs to be answered is how multiple PTMs can co-regulate RNP complexes [8,50], as was exemplified for the coordinated serine/threonine and tyrosine phosphorylation of FMRP and CAPRIN1, respectively [32]. To this aim, it will be invaluable to integrate protein-centric approaches with RNA-centric methods that can comprehensively characterise RNP assemblies on specific transcripts [51]. For example, multiplexing CLIP workflows [52,53] in conjunction with crosslinking mass spectrometry [54] presents one promising strategy to unravel simultaneous rearrangements of dozens of RBPs in response to signalling.

The experimental investigation of IDR phosphorylation in a cellular and organismic context poses a particular challenge (see Outstanding questions). To assess the functionality of phosphorylation sites, serines and threonines are usually replaced by phosphomimetic mutations, such as aspartate and glutamate. However, these are much less electronegative than a phosphoryl group and have a smaller hydration sphere and volume [55]. A predictive coarse-grained model was used to show that phosphorylation is expected to have a stronger effect on IDRs than phosphomimetic mutations [56]. A more sophisticated approach involves the site-specific incorporation of phosphorylated amino acids using an expanded genetic code [57,58] or their direct incorporation in live cells, which, however, remains challenging as these enter cells poorly and thus their excessive amount is required [59]. In particular, the recent finding that protein-RNA interactions can differ between cultured cells and mammalian organs emphasises the need to study regulatory mechanisms in the context of the organism [60].

In summary, phosphorylation of IDRs can affect RNA binding and function of RBPs in multiple ways, and often quite selectively. Recent studies have charted the way to uncovering the role of PTMs in shaping various aspects of protein-RNA interactions that determine signalling-dependent changes in gene expression.

## Figures and Tables

**Figure:**
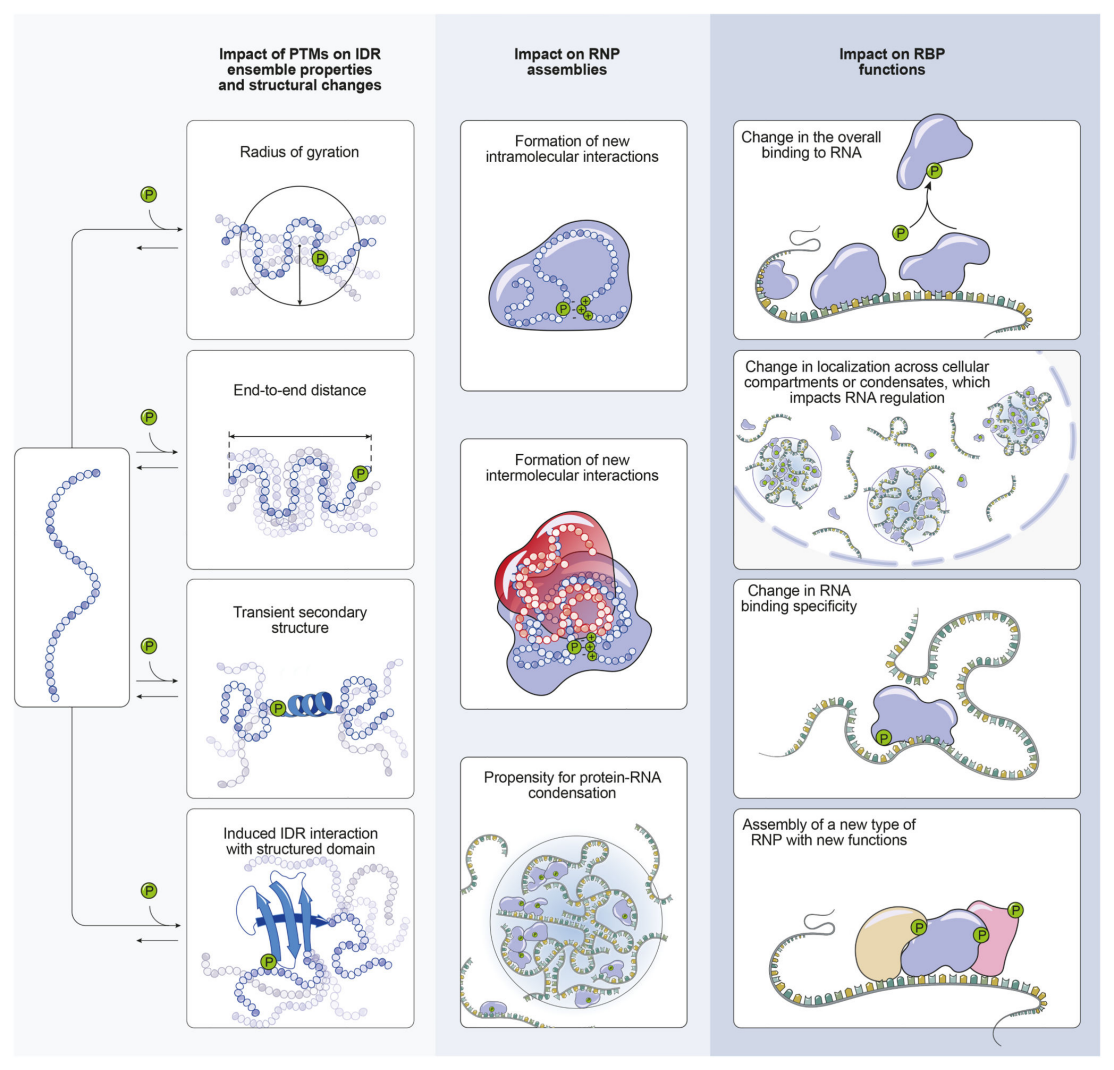
Phosphorylation of IDRs can affect an RNA-binding protein at various levels. In the left column we see possible effects of phosphorylation on the properties of the IDR ensemble. These effects can subsequently affect the interactions and condensation propensity that coordinate RNP assembly (centre column). Finally, changes in interaction and condensation propensity affect the regulatory functions of RBPs on specific RNAs (right-hand column). In the centre and right-hand columns, the IDR-containing RBPs are represented by blobs, whose variety of shapes indicates the malleability of the IDR conformational ensemble.

## Highlights

Many intrinsically disordered regions (IDRs) can be phosphorylated

Phosphorylation of IDRs can change their conformations and functions

Structural rearrangements of IDRs can influence the interaction of proteins with RNA

IDR phosphorylation can selectively alter global protein-RNA binding profiles

## Outstanding questions

- How does IDR phosphorylation selectively impact on RNA binding profiles of proteins?
- Can signalling coregulate assembly and condensation of multiple proteins on specific RNAs?
- How do multiple sites of post-translational modifications (PTMs) act together to modulate functions of an RNA binding protein (RBP)?
- How do PTMs modify biological functions of RBPs in the cellular and organismal context?

## References

[1] Kretova M (2023). Regulation of Pre-mRNA Splicing: Indispensable Role of Post-Translational Modifications of Splicing Factors. Life.

[2] England WE (2022). An atlas of posttranslational modifications on RNA binding proteins. Nucleic Acids Res.

[3] Vieira-Vieira CH (2022). Proteome-wide quantitative RNA-interactome capture identifies phosphorylation sites with regulatory potential in RBM20. Mol Cell.

[4] Zhao B (2021). Intrinsic Disorder in Human RNA-Binding Proteins. J Mol Biol.

[5] Walsh CT (2005). Protein posttranslational modifications: the chemistry of proteome diversifications. Angew Chem Int Ed Engl.

[6] Moses D (2024). Structural biases in disordered proteins are prevalent in the cell. Nat Struct Mol Biol.

[7] Darling AL, Uversky VN (2018). Intrinsic Disorder and Posttranslational Modifications: The Darker Side of the Biological Dark Matter. Front Genet.

[8] Bludau I (2022). The structural context of posttranslational modifications at a proteome-wide scale. PLoS Biol.

[9] Ottoz DSM, Berchowitz LE (2020). The role of disorder in RNA binding affinity and specificity. Open Biol.

[10] Jin F, Gräter F (2021). How multisite phosphorylation impacts the conformations of intrinsically disordered proteins. PLoS Comput Biol.

[11] Moses D (2020). Revealing the Hidden Sensitivity of Intrinsically Disordered Proteins to their Chemical Environment. J Phys Chem Lett.

[12] Gao J, Xu D (2012). Correlation between posttranslational modification and intrinsic disorder in protein. Pac Symp Biocomput.

[13] Miao Y (2018). Phospho-regulation of intrinsically disordered proteins for actin assembly and endocytosis. FEBS J.

[14] Hendus-Altenburger R (2017). A phosphorylation-motif for tuneable helix stabilisation in intrinsically disordered proteins - Lessons from the sodium proton exchanger 1 (NHE1). Cell Signal.

[15] He Y (2015). Phosphorylation-induced Conformational Ensemble Switching in an Intrinsically Disordered Cancer/Testis Antigen. J Biol Chem.

[16] Kasahara K (2018). Phosphorylation of an intrinsically disordered region of Ets1 shifts a multi-modal interaction ensemble to an auto-inhibitory state. Nucleic Acids Res.

[17] Bah A (2015). Folding of an intrinsically disordered protein by phosphorylation as a regulatory switch. Nature.

[18] Alderson TR (2023). Systematic identification of conditionally folded intrinsically disordered regions by AlphaFold2. Proc Natl Acad Sci U S A.

[19] Smyth S (2022). Multisite phosphorylation and binding alter conformational dynamics of the 4E-BP2 protein. Biophys J.

[20] König I (2015). Single-molecule spectroscopy of protein conformational dynamics in live eukaryotic cells. Nat Methods.

[21] Holehouse AS, Kragelund BB (2023). The molecular basis for cellular function of intrinsically disordered protein regions. Nat Rev Mol Cell Biol.

[22] Hentze MW (2018). A brave new world of RNA-binding proteins. Nat Rev Mol Cell Biol.

[23] Zeke A (2022). Deep structural insights into RNA-binding disordered protein regions. Wiley Interdiscip Rev RNA.

[24] Ray D (2023). RNA-binding proteins that lack canonical RNA-binding domains are rarely sequence-specific. Sci Rep.

[25] Harrington L (2021). De novo design of a reversible phosphorylation-dependent switch for membrane targeting. Nat Commun.

[26] Whitson SR (2005). Solution structure of the symmetric coiled coil tetramer formed by the oligomerization domain of hnRNP C: implications for biological function. J Mol Biol.

[27] Kato M (2022). How do protein domains of low sequence complexity work?. RNA.

[28] Banani SF (2017). Biomolecular condensates: organizers of cellular biochemistry. Nat Rev Mol Cell Biol.

[29] Wu H, Fuxreiter M (2016). The Structure and Dynamics of Higher-Order Assemblies: Amyloids, Signalosomes, and Granules. Cell.

[30] Wang A (2018). A single N-terminal phosphomimic disrupts TDP-43 polymerization, phase separation, and RNA splicing. EMBO J.

[31] Tsang B (2019). Phosphoregulated FMRP phase separation models activity-dependent translation through bidirectional control of mRNA granule formation. Proc Natl Acad Sci U S A.

[32] Kim TH (2019). Phospho-dependent phase separation of FMRP and CAPRIN1 recapitulates regulation of translation and deadenylation. Science.

[33] Shin C, Manley JL (2004). Cell signalling and the control of pre-mRNA splicing. Nat Rev Mol Cell Biol.

[34] Choi S (2023). The implications of alternative pre-mRNA splicing in cell signal transduction. Exp Mol Med.

[35] Xiang S (2013). Phosphorylation drives a dynamic switch in serine/arginine-rich proteins. Structure.

[36] Slišković I (2022). Exploring the multifunctionality of SR proteins. Biochem Soc Trans.

[37] Yang P (2020). G3BP1 Is a Tunable Switch that Triggers Phase Separation to Assemble Stress Granules. Cell.

[38] Barnhart MD (2022). Phosphorylation of the smooth muscle master splicing regulator RBPMS regulates its splicing activity. Nucleic Acids Res.

[39] Malki I (2022). Cdk1-mediated threonine phosphorylation of Sam68 modulates its RNA binding, alternative splicing activity and cellular functions. Nucleic Acids Res.

[40] Qiu C (2023). Intra- and inter-molecular regulation by intrinsically-disordered regions governs PUF protein RNA binding. Nat Commun.

[41] Modic M (2023). Poised PABP-RNA hubs implement signal-dependent mRNA decay in development. Nat Struct Mol Biol.

[42] He S (2022). The nexus between RNA-binding proteins and their effectors. Nat Rev Genet.

[43] Yi H (2018). PABP cooperates with the CCR4-NOT complex to promote mRNA deadenylation and block precocious decay. Mol Cell.

[44] Webster MW (2018). mRNA Deadenylation Is Coupled to Translation Rates by the Differential Activities of Ccr4-Not Nucleases. Mol Cell.

[45] Hallegger M (2021). TDP-43 condensation properties specify its RNA-binding and regulatory repertoire. Cell.

[46] Gruijs da Silva LA (2022). Disease-linked TDP-43 hyperphosphorylation suppresses TDP-43 condensation and aggregation. EMBO J.

[47] Buratti E (2018). TDP-43 post-translational modifications in health and disease. Expert Opin Ther Targets.

[48] Gueroussov S (2017). Regulatory Expansion in Mammals of Multivalent hnRNP Assemblies that Globally Control Alternative Splicing. Cell.

[49] Ule J, Blencowe BJ (2019). Alternative Splicing Regulatory Networks: Functions, Mechanisms, and Evolution. Mol Cell.

[50] Sridharan S (2022). Systematic discovery of biomolecular condensate-specific protein phosphorylation. Nat Chem Biol.

[51] Hafner M (2021). CLIP and complementary methods. Nature Reviews Methods Primers.

[52] Lorenz DA (2023). Multiplexed transcriptome discovery of RNA-binding protein binding sites by antibody-barcode eCLIP. Nat Methods.

[53] Wolin E (2023). SPIDR: a highly multiplexed method for mapping RNA-protein interactions uncovers a potential mechanism for selective translational suppression upon cellular stress. bioRxiv.

[54] Graziadei A, Rappsilber J (2022). Leveraging crosslinking mass spectrometry in structural and cell biology. Structure.

[55] Newcombe EA (2022). How phosphorylation impacts intrinsically disordered proteins and their function. Essays Biochem.

[56] Perdikari TM (2021). A predictive coarse-grained model for position-specific effects of post-translational modifications. Biophys J.

[57] Luo X (2017). Genetically encoding phosphotyrosine and its nonhydrolyzable analog in bacteria. Nat Chem Biol.

[58] Beránek V (2018). Genetically Encoded Protein Phosphorylation in Mammalian Cells. Cell Chem Biol.

[59] Hoppmann C (2017). Site-specific incorporation of phosphotyrosine using an expanded genetic code. Nat Chem Biol.

[60] Perez-Perri JI (2023). The RNA-binding protein landscapes differ between mammalian organs and cultured cells. Nat Commun.

